# Pain Detection with Bioimpedance Methodology from 3-Dimensional Exploration of Nociception in a Postoperative Observational Trial

**DOI:** 10.3390/jcm9030684

**Published:** 2020-03-04

**Authors:** Martine Neckebroek, Mihaela Ghita, Maria Ghita, Dana Copot, Clara M. Ionescu

**Affiliations:** 1 Department of Anesthesia, Ghent University Hospital, Corneel Heymanslaan 10, 9000 Ghent, Belgium; Martine.Neckebroek@UGent.be; 2 Research group of Dynamical Systems and Control, Ghent University, Tech Lane Science Park 125, 9052 Ghent, BelgiumDana.Copot@UGent.be (D.C.); ClaraMihaela.Ionescu@UGent.be (C.M.I.); 3 EEDT—Core Lab on Decision and Control, Flanders Make Consortium, Tech Lane Science Park 131, 9052 Ghent, Belgium; 4 Department of Automatic Control, Technical University of Cluj Napoca, Memorandumului 28, 400114 Cluj-Napoca, Romania

**Keywords:** post-operative pain, bioimpedance, time-frequency analysis, analgesia, anesthesia, drug delivery control

## Abstract

Although the measurement of dielectric properties of the skin is a long-known tool for assessing the changes caused by nociception, the frequency modulated response has not been considered yet. However, for a rigorous characterization of the biological tissue during noxious stimulation, the bioimpedance needs to be analyzed over time as well as over frequency. The 3-dimensional analysis of nociception, including bioimpedance, time, and frequency changes, is provided by ANSPEC-PRO device. The objective of this observational trial is the validation of the new pain monitor, named as ANSPEC-PRO. After ethics committee approval and informed consent, 26 patients were monitored during the postoperative recovery period: 13 patients with the in-house developed prototype ANSPEC-PRO and 13 with the commercial device MEDSTORM. At every 7 min, the pain intensity was measured using the index of Anspec-pro or Medstorm and the 0–10 numeric rating scale (NRS), pre-surgery for 14 min and post-anesthesia for 140 min. Non-significant differences were reported for specificity-sensitivity analysis between ANSPEC-PRO (AUC = 0.49) and MEDSTORM (AUC = 0.52) measured indexes. A statistically significant positive linear relationship was observed between Anspec-pro index and NRS (r^2^ = 0.15, *p* < 0.01). Hence, we have obtained a validation of the prototype Anspec-pro which performs equally well as the commercial device under similar conditions.

## 1. Introduction

Pain is considered a subjective feeling (described by emotional aspects that influence pain assessment scales) with objective roots (represented by the biological processes along the pain pathway) [1]. Then, its management is generally based on assessment of the human body’s reaction to noxious stimulation [2]. Since nociception is defined as the physiological encoding and processing of nociceptive stimuli [3], the frequency modulation of the measured signal from the body should be considered.

Intraoperative analgesia or postoperative pain management requires to optimally be driven by an objective assessment of the patient’s status, for avoiding the complication of intraoperative awareness that continues to occur or for limiting the negative impact on postoperative hospital length of stay and costs [4,5]. Several bio-markers have been studied in order to monitor the nociception level, but these parameters lack an analysis in both time and frequency domain [6]. Consequently, we have recently developed a device for pain assessment, namely ANSPEC-PRO. The prototype has been validated on awake healthy volunteers undergoing induced pain, with successful results in detecting and characterizing perception of pain [7]. The method is based on the detection of nociceptor stimulation in the frequency-modulated skin impedance via non-invasive measurement in hand palm.

Although several commercially devices [2,8,9], as well as newly researched principles [10,11], are currently available and clinically evaluated in many trials, no monitor is widely and routinely used for analgesia assessment [12]. Hence, what differentiates Anspec-pro from the other available devices is the mathematical formalism for parametric modelling of time-frequency variations of skin impedance values [13]. Already developed methods show that changes in specific frequency ranges have the ability to characterize the cortical activity (e.g., gamma frequencies of EEG signal by the dimensionless index, WAV_CNS_ [14,15]) or the parasympathetic nervous system (e.g., high-frequency heart rate variability index [16]). Moreover, skin conductance is a long-known tool for quantifying the sympathetic nervous system changes determined by immediate effect of pain, but not influenced by hemodynamic variability [17]. Time-frequency scanning is a novel aspect that has not been yet assessed for the skin-extracted electrical signals in nociception detection, but ongoing validation should soon clarify its medical relevance. In addition, it has already been shown that mathematical formulation is an important tool in characterization of drug tissue trapping [18]. The rationale of using bioimpedance spectroscopy is that it allows a rigorous characterization of the biological tissue electrical properties influenced by nociception [19,20]. The in-house developed prototype and methodology have the advantage to follow a customizable input signal that excites the tissue over a range of frequencies.

The aim of this pilot study is to validate our impedance-based methodology for postoperative pain evaluation. We primarily test the hypothesis whether the nociception measured by Anspec-pro is correlated with the standard tool (i.e., numerical rating scale—NRS). Our secondary hypothesis is that the specificity and sensitivity of Anspec-pro is similar to an existing device based on a similar approach (skin conductance measurement), namely MEDSTORM. The skin sympathetic nervous system and emotional sweating, mirrored by skin conductance response have been previously reported to assess pain in patients, post-operatively [21] as well as perioperatively [22]. The patients for this trial were equally distributed and randomly assigned to be evaluated with one of the two devices.

As such, the focus of the study is not to perform a comparison analysis between the developed prototype and the commercial device. We need to validate Anspec-pro with an already approved device that is based on similar methodology, under the same conditions. On long term, our methodology will enable evaluation of pain by means of mathematical models which can be then included in a fully automated drug delivery for anesthesia system [23,24,25,26,27]. Thus, the secondary objective of this observational trial is frequency exploration of the tissue electrical properties influenced by nociception processes. The present method could offer a promising technique for accurate assessment of pain in terms of impedance.

## 2. Experimental Section

### 2.1. Study Design

The prospective observational study was approved by the Ethics Committee of Ghent University Hospital (Protocol code: EC/2017/1517). The study has been registered before patient enrolment at clinicaltrials.gov (Identifier: NCT03832764; principal investigator: M. N.; date of registration: 6 February 2019). This manuscript adheres to the applicable recommendations of Strengthening the Reporting of Observational studies in Epidemiology (STROBE) statement [28].

Written informed consent was obtained from each patient during their preoperative consultation with the anesthesiologist. The course on Good Clinical Practice (GCP) has been completed by the sponsor and each technician (also authors) that were operating the devices during this clinical trial for data collection.

### 2.2. Patients

The trial was a cohort study performed between 24 April 2018 and 22 June 2018 at Ghent University Hospital, Belgium. The patients included in the study were randomly chosen from the list of the patients that were planned to have a surgical operation under general anesthesia, considering inclusion/exclusion criteria approved through the protocol. The principal investigator evaluated the eligibility for the patient’s enrolment, informed the prospective patient about the trial, and obtained the informed consent. The patient allocation was made following simple randomization procedure to 1 of the 2 monitoring devices, using sealed opaque envelopes. On the day of surgery, the principal investigator assigned the participants by asking them to choose between 26 identical envelopes in which the Anspec-pro or the Medstorm choice was inside (13 envelopes with Anspec-pro and 13 envelopes with Medstorm). Patients were informed about the result and saw the monitor before starting the procedures.

The inclusion criteria were as follows: (i) age between 18 and 75 years old, (ii) patients able to comprehend, sign and date the written informed consent document to participate in the clinical trial, (iii) patients planned for a surgical procedure under general anesthesia and expected to need a strong analgesic, type opioid, like sufentanil, during the operation and (iv) American Society of Anesthesiologists (ASA) physical status Class I–III classified by the anesthesiologist.

The exclusion criteria were as follows: (i) patients having epidural analgesia infused by a pain pump, (ii) patients with chronic pain or getting medication used for chronic pain, like antiepileptics, antidepressants, opioids; (iii) participation in a clinical trial within the past 30 days; (iv) operation planned at one of the upper limbs (so that placing together the blood pressure cuff and the electrodes of the pain monitor at the same upper limb would not be possible), (v) patients staying in daily hospital or in Intensive Care Unit and (vi) pregnant women (the patient was asked before the operation). It was stated that subjects must have been withdrawn under the following circumstances: (i) at their own request, (ii) if the investigator feels it would not be in the best interest of the subject to continue, and (iii) if the subject violates conditions laid out in the consent form/information sheet or disregards instructions by the clinical investigation personal.

### 2.3. Study Protocol

The surgical schedule of Ghent University Hospital was assessed in order to determine subjects who met the criteria for study enrolment. During all the three phases of the study (pre, intra, and post-operative), all patients received care after local standards in all groups and the routine monitoring was made. Care providers did not make changes to the used anesthetics based on the outcomes of Medstorm or Anspec-pro devices.

**Figure 1:**
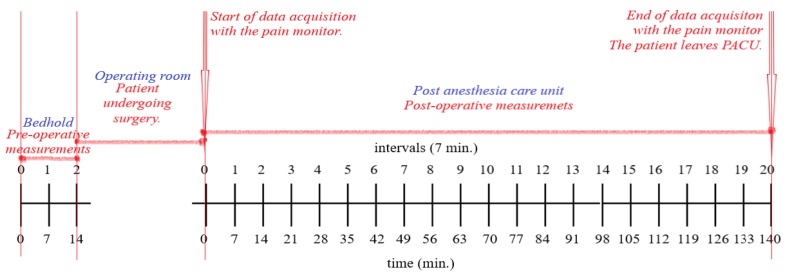
Clinical trial protocol used for Anspec-pro device and Medstorm measurements for pain monitoring.

The study protocol consisted of two periods of pain observation: (1) preoperative: in the waiting room the patient’s pain was assessed by NRS (self-report) and also by the pain monitor for 14 min; this data was used as a baseline for the measurements after the surgery; (2) postoperative: in Post-Anesthesia Care Unit (PACU), the pain was measured like usual, thus by NRS (self-report or nurse evaluation at every 7 min), but also by the pain monitor, continuously for 140 min. The measurement protocol is depicted in Figure 1.

All patients of the two study groups had a general anesthesia during surgery. The performed procedures were abdominal, gynecology, otorhinolaryngology, and urology. The general anesthesia was done like standard care and the anesthetists chose the anesthetics and analgesics during the operation. All medications were registered in the digital medical file of the patient. No changes to the study protocol were made after trial commencement.

### 2.4. Outcomes

The primary outcome parameters were the postoperative pain values, namely the Index of Medstorm, the Index of Anspec-pro, and NRS. In the statistical analyses, the indexes of Medstorm or Anspec-pro were considered dependent variables predicted from NRS values.

Secondary endpoints were the vital parameters and the ratings of subjective conditions (vigilance, well-being, energy level, agitation, and nausea).

Postoperative data collection included other candidate variables considered for statistical analysis: mean heart rate (HR) and non-invasive mean arterial pressure (MAP).

The subjective conditions during the study have been reported for being considered in discussions and interpretation of results: GCS score (Glasgow coma scale), the movement of the patient, the location of the pain monitor electrode (left/right hand), all medications including dose and time of administration (pre-, peri-, and post-operative), medical and surgical history of the patient. The medications were important to be documented in order to further correlate them in future analysis with time-frequency changes in the measured impedance signal.

### 2.5. Data Collection

Each variable of interest has been measured and collected through the same protocol. Data were derived from four sources: (1) the nociception index from the pain monitor (Anspec-pro or Medstorm), (2) the NRS value from the patient (if totally awake) or from the nurse, (3) vital parameters (blood pressure, heart rate, oxygen saturation) from the electronic medical record, (4) every real-time observation that could influence the measurements from the device’s technician or nurse.

For tracking NRS, the patients were asked to quantify their level of pain on a numeric rating scale (0–10). For the patients not fully conscious, the nurses reported the NRS value assessed for the patient based on their experience.

Standard care and medication were given like usual pre-, peri-, and postoperative, estimated by the experience of the nurse from recovery room or the anesthesiologist. The choice of the pain monitor was done by randomization, after the patient agreed to be part of the study.

Before the operation, the index from the pain monitor (after randomized selection), the NRS and the vital parameters have been continuously monitored for 14 min. The values of these variables were registered at each 7 min (e.g., minutes 0, 7th, and 14th). The time interval has been selected together with the clinical investigator as an optimal interval for pain assessment. Hence, the choice of 7 min is also a trade-off between the limitations given by the technology and the clinically applicable interpretation. After this step, the surgery was performed according to normal clinical procedures, while the patient’s pain was not monitored with the pain device; the perioperative medications given to the patient were noted in the personal file. After the operation, the patient stayed in the recovery room, getting the standard care from nurses. To determine the correlation between the index given by the pain monitor and the NRS value, all these parameters have been continuously monitored for 140 min. Thus, at each 7 min, the index from the pain monitor, the NRS, as well as the vital parameters were noted (21 measurements in total, with the first one being measured in the first minute after the patient was connected at the pain monitoring device in the recovery room).

The primary endpoints (pain level) were measured using the randomly assigned nociception monitoring systems and NRS.

### 2.6. Monitors Used for Data Collection

One monitor was MED-STORM PAIN MONITOR^TM^, ref: MD1001 (CE0413, Med-Storm Innovation, AS, Oslo, Norway), which calculates the Skin Conductance Algesimeter [29]. The monitor generates the number of fluctuations of skin conductance per second (NFSC) [17]. For this clinical trial, all the procedures followed the instructions from Medstorm User Manual (Version 1.0 English) and the data collection methods used in previous studies of NSCF in the postoperative setting [21]. Incoming data were displayed on-line on a laptop connected to the monitor, on which the provided software from manufacturer was previously installed. Data was collected and automatically analyzed in application mode “Postoperative”. The manufacturer’s electrodes were placed at the hand palm of the patient and connected with the monitor. The index NFSC is correlated by the manufacturer with NRS or VAS (Visual Analogic Scale) score: the sensitivity to determine pain less than 3 or like 3 on the NRS/VAS has the index less than or like 0.21 (peaks per second); the sensitivity to determine pain in the range 3–5 on NRS/VAS scales has the index between 0.21–0.27 (peaks per second); the sensitivity to determine pain in the range 6–10 on NRS/VAS scales, has the peaks per seconds more than 0.27.

**Figure 2:**
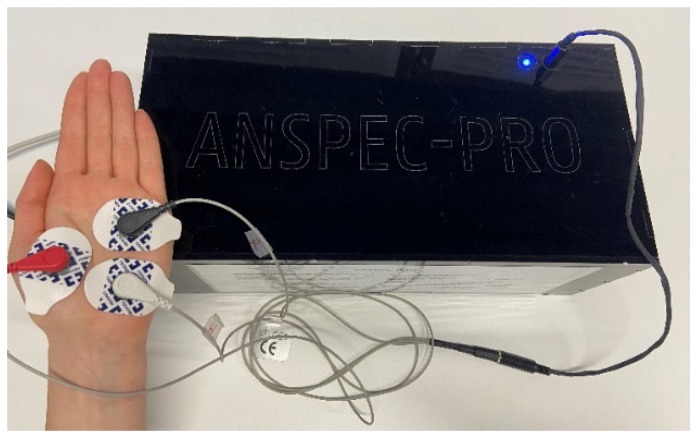
ANSPEC-PRO measurement device and electrodes placement.

The other monitor was ANSPEC-PRO (Ghent University, Ghent, Belgium—only for research use), a new non-invasive pain monitor evaluated for the first time in a clinical trial (Approval FAMHP no.: 80M0707, 03/04/2018). It has been previously validated on awake participants with self-induced nociceptor excitation, after informed consent was given [7,30]. Anspec-pro injects in the skin a current containing multiple frequencies via electrodes and measures the bio-modulated response voltage. The proposed method is based on the fact that frequency affects the pain detection because each tissue molecule has a different response to a given frequency, based on its electrical permittivity. This permittivity decreases in three main steps corresponding to three dispersions: α: 10 Hz–10 KHz characteristic for diffusion detection of the ionic species (extracellular fluid level), β: 10 KHz–10 MHz for dielectric property measurement of the cell membrane, and γ > 10 MHz for content measurement of the biological species (intracellular fluid level) [31]. Since pain is an electrical signal transmitted by dynamics of ions signaling among intra- and extra-cellular fluid, the lowest frequency range was firstly used. The diffusion of sodium/potassium ions changes the typical extracellular concentration, resulting in a negative charged extracellular fluid, so an action potential can be seen as variations of the electrical impedance of the tissue over the nociception pathway. The methodology for the prototype development and measurements are detailed in [13]. The measurement requires three-point electrodes applied on the palmar skin, as illustrated in Figure 2. The monitor is connected to a laptop and the collected data is post-processed in order to generate the complex impedance using spectral analysis [30]. The aim of calculating the impedance is to obtain a pain index that will be further correlated with the NRS given by the patient at every 7 min. The pain index is calculated as the mean of impedance values over 7 min, where the impedance from each minute is also the mean of the values obtained from each frequency (the device measures an output signal at 29 different frequencies, obtaining 29 values). The result is an absolute value of the impedance, correlated with each BMI’s (body mass index) patient (the considered index of Anspec-pro).

Both devices Medstorm and Anspec-pro are non-invasive pain monitors. Monitoring was not blinded so that the observers could see the data collected curves and note when artifacts disturbed the registration. When the signal quality was distortional as a result of change of the integrity of the measured signal or temporary loss of connection, the event was registered as “artifact”, as were situations when the patient moved their measurement hand where the sensors were applied. In the case of Medstorm, the device’s software automatically decides if the signal quality was bad.

### 2.7. Potential Bias Reduction

Both nociception monitors measure bio-electrical data at the hand level, typically accounted as the appropriate location to detect the micro-fluctuations in skin electrical permeability (caused by the fill of plantar sweat glands under sympathetic control). Additionally, the post-operatory level of pain can be influenced by the level of traumatology of the surgery. Consequently, we carefully considered the type of intervention, including the part of the body concerned for the surgical procedure, as potential sources of bias. Measures were taken to reduce bias by selecting patients for enrolment that were planned for similar procedure types.

### 2.8. Statistical Methods

Given the fact there was no comparable previous study (that includes Anspec-pro monitor), this was planned as a pilot trial.

Statistical analysis was performed using Matlab Software (R2017a). Values are presented as number of patients *n* (%), medians (interquartile ranges—IQR (range)) or mean (standard deviation—SD). Analysis of variance (ANOVA) was used for accessing the intergroup differences between the outcomes of the study.

The relationship between two variables was described by two methods: correlation and regression analysis. The correlation analysis was performed based on the covariance as a measure of how much two random variables vary together. The values of the correlation coefficients (r) can range from −1 to 1, with −1 representing a direct, negative correlation, 0 representing no correlation, and 1 representing a direct, positive correlation. Linear regression was used to investigate if one variable can be predicted from another, by assessing the coefficient of determination (r^2^). A receiver-operating characteristic (ROC) curve was built by plotting the sensitivity, or true positive rate, as a function of the false positive rate (1-specificity). The area under a ROC curve (AUC) represents the predictive ability of the test and should be at least 0.75 for good prediction. Statistical significance was accepted for *p* < 0.05.

## 3. Results

**Figure 3:**
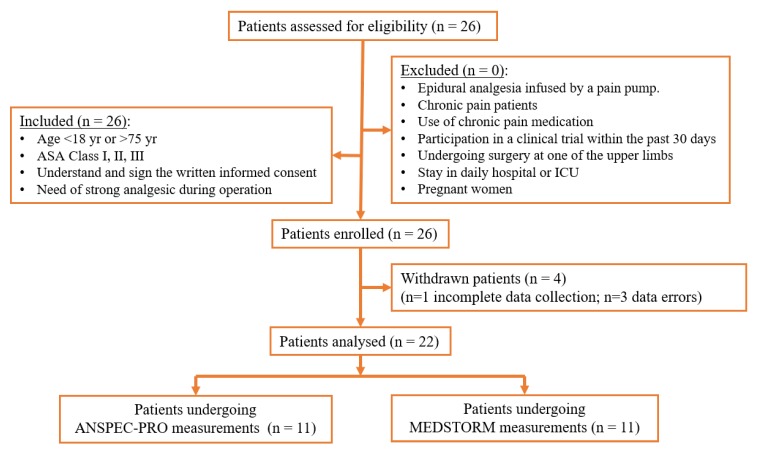
Study subject flow diagram.

The principal investigator screened 26 patients scheduled for different surgeries under general anesthesia in the operation theatre of Ghent University Hospital. A flow diagram denoted in Figure 3 shows the details of assessment and exclusion. Twenty-six patients were enrolled in the study; data from four patients was excluded because: for one patient the measurement was interrupted during the post-anesthesia monitoring and data missed; for three patients, the data enregistered artefacts.

For statistical analysis, data from 22 patients was included. Subject demographics and characteristics are shown in Table 1. Age, height, weight and BMI were not significantly different (*p* > 0.2) between the two groups.

The postoperative clinical data acquired in PACU is summarized in Table 2. The mean of each pain index was not considered for analysis, having non-comparable magnitudes. The methods of calculation for each pain index are different, resulting in an absolute value of the complex impedance as a function of frequency (Anspec-pro method) and a number of skin fluctuations per second (Medstorm method). The NRS score (median (interquartile range (range))) was significantly higher in the Anspec-pro group than in Medstorm (4 (2–6 (0–9)) versus 2 (0–3 (0–8)), *p* < 0.001). In addition, the vital parameters (MAP [mmHg], HR [beats·min^−1^] were significantly lower in Anspec-pro group compared with Medstorm group (*p* < 0.001).

The correlation analysis showed no significant relationship between the pain indexes from devices and NRS, neither in the Anspec-pro group nor in the Medstorm group (correlation coefficient r = 0.39 in Anspec group vs. r = −0.066 in Medstorm group, *p* = 1). No significant correlation was found between Anspec-pro index and HR (r = 0.068, *p* = 1) or Anspec-pro index and MAP (r = 0.13, *p* = 1). The correlation was statistically non-significant as well between Medstorm index and HR (r = 0.29, *p* = 1) or Medstorm index and MAP (r = 0.04, *p* = 1).

**Figure 4:**
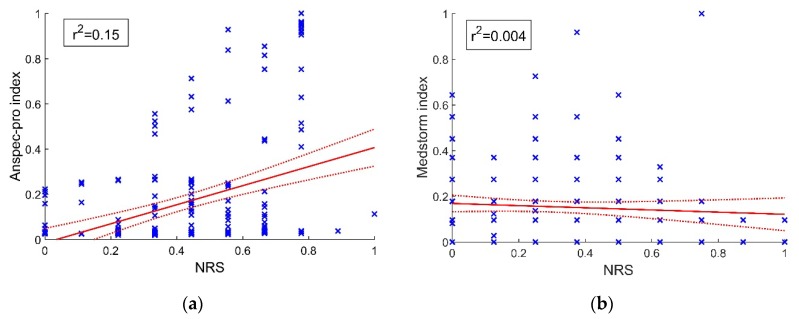
Linear data fitting and confidence bounds: (**a**) positive linear relationship between Anspec-pro index and numeric rating scale (NRS) score; (**b**) non-significant linear relationship between Medstorm index and NRS score. The presented data is normalized. NRS—Numerical Rating Scale.

A statistically significant positive linear relationship (Index_Anspec-pro_ = −0.58 + 1.8018 × NRS, r^2^ = 0.15, *p* < 0.01) was observed between Anspec-pro index and NRS during the stay in PACU, with 15% of the variance in Anspec-pro index predicted by NRS, as seen in Figure 4a. On the other hand, the linear relationship between Medstorm index and NRS assessed during PACU observations had no statistical significance (Index_Medstorm_= 0.12 − 0.004 × NRS, r^2^ = 0.004, *p* = 0.328), as depicted in Figure 4b.

**Figure 5:**
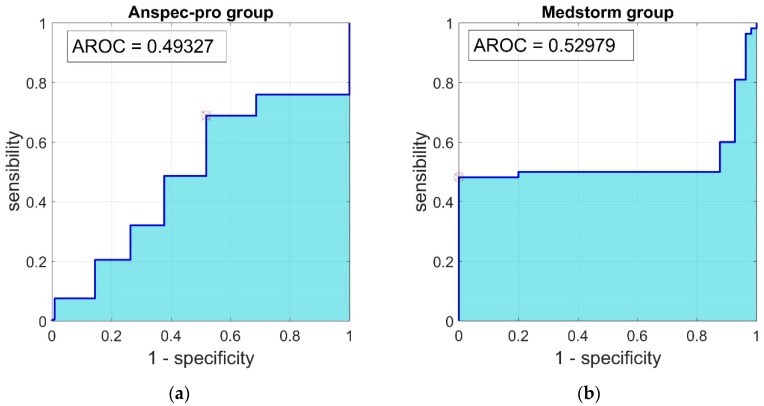
Receiver-operating characteristic (ROC) curve for the relationship between sensitivity and 1–specificity, determining the performance of (**a**) Anspec-pro index, (**b**) Medstorm index, to predict postoperative pain (NRS). AUC—Area Under the Curve; ROC—Receiver-Operating Characteristic.

The ROC curve determining the performance of Anspec-pro index respectively of Medstorm for predicting NRS is shown in Figure 5. The sensitivity and specificity of Anspec and Medstorm methods were similar, as the area under the curve for Anspec-pro (AUC = 0.49) was non-significantly different from the one of Medstorm device (AUC = 0.52).

**Figure 6:**
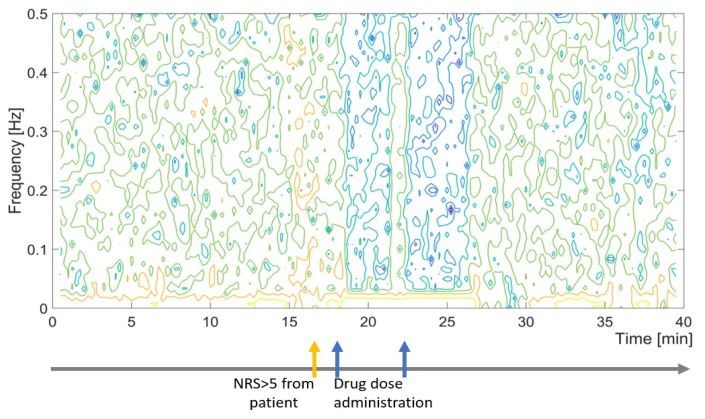
Sample spectrogram and log events for measured impedance values with ANSPEC-PRO for one patient during first 40 minutes in PACU.

Spectrographic analysis of the skin impedance measured with ANSPEC-PRO device was approached to obtain a wider view towards the signal [32]. Based on the significant frequency components present in the original time-based signal, the power spectral density (PSD) was returned for normalized frequencies. The spectrogram presented in Figure 6 denotes the PSD of the measured skin impedance signal for one patient chosen as an example. The PSD energy plot suggests that during the first 40 minutes in PACU, the bioimpedance changes its main energy content (changes in color intensity), related to the pain events and medication administration. The nonlinearity of PSD over time can originate from the differences of tissue electrical states determined by the generation and inhibition of nociception events.

## 4. Discussion

The results of this pilot study demonstrate that non-significant differences were reported for specificity-sensitivity analysis between ANSPEC-PRO and MEDSTORM measured indexes. Hence, we have obtained a validation of the prototype Anspec-pro which performs equally well as the commercial device under similar conditions. The AUC for Medstorm was 0.52 and for Anspec-pro it was 0.49. The correlation analysis showed no significant relationship between the pain indexes from devices and NRS, neither in the Anspec-pro group nor in the Medstorm group (with correlation coefficient r = 0.39 respectively r = −0.066). In addition, no significant correlation was found between Anspec-pro index or Medstorm index and the monitored vital parameters (HR and MAP). Even small, a statistically significant 15% of the variance in Anspec-pro index was predicted by NRS, while no statistically significant linear relationship was observed between Medstorm index and NRS (r^2^ = 0.004). Although the NRS is the golden standard nowadays to measure pain, we see that the ROC-curve for the two monitors is not conclusive, perhaps since both are sensitive to artefacts (e.g., movements). Further adaptation of the software according to the physiological parameters, like including an NRS-guided algorithm may be an interesting next step.

Artificial intelligence has been advancing in anesthesiology, including depth of anesthesia monitoring, control infusion, or pain management [33]. However, despite decades of research on optimal pain management, postoperative pain continues to be inadequately treated [34,35]. The almost ubiquitous acute pain after surgery that mostly resolves within one week can persist beyond the usual time of recovery and transform into a chronic pain state [36]. Major causes of this problem are inadequate pain assessment and lack of clear guidance [37]. Globally, the massive volume of surgery represents a vast potential for postsurgical pain, with a rise from 234 million operations (2004) to 313 million (2012) [38]. Therefore, analgesia monitoring that provides well-tolerated pain relief after surgery is of utmost importance.

Besides biomarkers for pain detection under general anesthesia [16], modelling of physiological nociception and drug pharmacokinetics/pharmacodynamics (PK/PD) have a significant impact on anesthesia drug administration [39,40]. The performance of the traditional target-controlled infusion systems for anesthesia can be improved by the real-time adaptation of PK/PD models [41,42,43]. This is provided by the closed-loop feedback systems that integrate the pharmacological and effect modelling [44,45,46,47]. However, the drug-dose relationship for analgesia still lacks a direct PD model to a pain monitor, propofol and remifentanil being mostly linked to BIS (Bispectral index) [42,48,49]. This can be represented by a Hill curve using the output from the commercial devices or our prototype, while the last one enables the use of transfer function to evaluate the analgesia state of the patients more in detail (e.g., by using the frequency response).

To measure is to know. This is a very well understood concept across all disciplines. Frequency response allows one to determine approximate models for the process at hand. These procedures usually require multiple or persistent exciting tests with input signals of various frequencies, so that the frequency response can be estimated over the required frequency range [50]. In this particular case, the essential benefit of the ANSPEC-PRO device is that it allows a customizable input signal to the system under observation. In the particular studies case, the ANSPEC-PRO sends a composed multisine with optimal crest factor for optimal extraction of dynamic process information for identification purpose of a process model [13]. MEDSTORM, by contrast, has a single sine input at a single frequency, and the recorded signal back from the skin impedance is not processed as a function of frequency (due to its single point data) but in the time domain, using a moving window averaged amplitude correlated with the pain and awareness in the patient. The disadvantage is that at the cost of the same measuring principle, a great deal of opportunity to gather more information without any extra cost for the patient remains unexplored. It is here that the Anspec-pro excels, i.e., it has a multiple point in frequency signal recording data, thus allowing extraction of a dynamical frequency response which can be evaluated also as a function of time, i.e., a time-frequency analysis of the skin impedance to be correlated to pain levels. Our future publications will present the frequency analysis of nociception from a more engineering-centered point of view.

It is not our claim as to whichever device is better. Since they both use the same measuring principles and sensors (i.e., skin impedance data), the difference is more intrinsically in the amount of data collected and post-processing. We hypothesize that the skin impedance is a dynamic system, where dynamic processes occur as a function of noxious stimulation and of opioid effects. By definition of system engineering principles of identification, characterization of a dynamic process requires a persistent excitation: in our case the multisine signal composed of sum of sinusoids in a bandlimited frequency interval. This allows to extract a transfer function of the process, i.e., a function which characterizes the dynamical processes taking place. A transfer function can never be defined by a single frequency point, as is the case with MEDSTORM. Hence, with ANSPEC-PRO, the user has more information to process, with a prospective higher degree of usefulness for clinical research. It was not our purpose to propose yet another pain monitor on the market, but rather to introduce the rationale and the means to the clinical research community for performing more in-depth analysis of the complex pain pathway monitoring process.

Our study presents, however, some limitations. Factors such as postoperative cofounders (i.e., movements, anxiety) influence skin electrical properties in awake subjects. Additionally, our results may not be extrapolated to any category of patients (i.e., different analgesic drugs have been used without an effect analysis of these drugs on the monitored pain index).

## 5. Conclusions

In conclusion, we merely wanted to verify that the proposed versatile instrumentation delivered by ANSPEC-PRO and methods used to extract information via skin impedance have meaningful clinical results. This assumption has been successfully verified as it delivers similar results to a commercial device, i.e., MEDSTORM. Through the availability of the ANPSEC-PRO instrumentation and methodology, there is a great opportunity to push-through technology and clinical state of art for pain monitoring and related research. Future research opportunities are related to the biomedical engineering domain through the analysis of the frequency response function which better characterizes the nociception dynamical process.

## Figures and Tables

**Table 1:** Patients characteristics and clinical data.

Characteristic	ANSPEC-PRO (*n* = 11)	MEDSTORM (*n* = 11)
Biometric data		
Age (y)	34.90 (12.46)	41.54 (18.26)
Height (cm)	167.45 (10.01)	173.72 (12.58)
Weight (kg)	69.72 (13.38)	74.18 (17.41)
BMI (kg·cm^−2^)	24.81 (4)	24.24 (3.2)
Gender *n* (%)		
Male	1 (0.09)	6 (0.54)
Female	7 (0.63)	4 (0.36)
Transman	3 (0.27)	1 (0.09)
Surgery type *n* (%)		
ORL	3 (0.27)	5 (0.45)
Abdominal	2 (0.18)	1 (0.09)
Gynecology/Urology/Orthopedics	5 (0.45)	5 (0.45)
Breast surgery	1 (0.09)	0
ASA class *n* (%)		
I	3 (0.27)	4 (0.36)
II	8 (0.72)	6 (0.54)
III	0	1

Values are means (SD)—95% confidence intervals)—respectively counts (%) from total number of patients of the group monitored with the same device; BMI—Body Mass Index; ORL—Otorhinolaryngology; ASA—American Society of Anesthesiologists physical status.

**Table 2:** Postoperative outcome parameters in post-anesthesia care unit (PACU).

Variables	ANSPEC-PRO GROUP (*n* = 11)	MEDSTORM GROUP (*n* = 11)	*p*-Value
Pain index from device	1.31 (1.14–6.56 (0.71–38.48))	0.07 (0–0.2 (0–0.73))	<0.001
NRS	4 (2–6 (0–9))	2 (0–3 (0–8))	<0.001
HR [Beats/min]	68 (59–77.5 (40–99))	74.5 (65–84 (46–107))	<0.001
MAP [mmHg]	86.66 (78.66–93.5 (63–113))	91.66 (84.66–97.83 (72–116))	<0.001

Values are median (IQR (range)) from total number of patients of the group monitored with the same device; PACU—Post-Anesthesia Care Unit; NRS—11 points Numeric Rating Scale (0–10); HR—Heart Rate; MAP—Mean Arterial Pressure.
